# Supplementary material for: Content-rich biological network constructed by mining PubMed abstracts
Source: BMC Bioinformatics. 2004 Oct 8;5:147. doi: 10.1186/1471-2105-5-147 (PMC528731; doi:10.1186/1471-2105-5-147)
Supplement: Additional File 2 — The original results of the above study (non-essential files are deleted to keep the file size under the limit set by BMC bioinformatics). [file 1471-2105-5-147-S2.bz2 › chilibotAdditionalFile2/dip20/604ID9729040E10983/html/H2_B2M.html]

 


 **H2** and **B2M** 
  
Found 20 abstracts in PubMed, retrieved 20.  
 

 What does Google say? 
 PDF only 
| .edu only 

---

**Interactive relationship** (e.g. stimulation, inhibition, etc)

**Inhibitory relationship**- Although these cells express the CD4 co receptor, they are present in  **H2**  1 A beta A beta gene disruption mutants but are markedly reduced in beta 2 microglobulin  [ **B2M** ]  beta 2m mutant mice and hence are beta 2m dependent.  Ref: 8580071 Int Immunol, 1995
- Incubation of T lymphocytes or TR lymphocytes with adenosine or impromidine, an  **H2**  histamine agonist, under conditions previously shown to increase Fc gamma receptors and radioresistant suppressor cell activity, was found to increase the proportion of cells expressing readily detectable surface beta 2 microglobulin  [ **B2M** ]  and the antigen detected by OKT8.  Ref: 6177462 Clin Exp Immunol, 1982
**Neutral relationship**- Expression of each HLA hyb molecule and HLA B27 nat human beta 2 microglobulin  [ **B2M** ]  led to peripheral CD8 T cell levels comparable with that for mice expressing a single  **H2**  K b or  **H2**  D b gene.  Ref: 12421934 J Immunol, 2002
- These data indicate that the expression of T lymphocyte Fc gamma receptors, beta 2 microglobulin  [ **B2M** ]  and the antigens detected by the monoclonal antibodies OKT4 and OKT8 are, at least in part, regulated by agents acting upon adenosine and  **H2**  histamine receptors.  Ref: 6177462 Clin Exp Immunol, 1982

**Non-interactive relationship** (e.g. studied together, co-existance, homology, etc.)

- In addition, these preliminary studies indicated that there was little or no beta 2 microglobulin  [ **B2M** ]  beta 2 mu in the immunoprecipitates with the  **H2**  2Db heavy chain.  Ref: 7005347 J Immunol Methods, 1980
- We previously described Tg mice carrying the HLA B27, HLA B7, or HLA A2 alleles expressed as fully native HLA nat with human beta 2 microglobulin  [ **B2M** ]  and as hybrid human mouse HLA hyb molecules on the  **H2**  b background.  Ref: 12421934 J Immunol, 2002
- IFN type II treatment of w.t. and IFN alpha beta resistant cell lines results in an increased expression of  **H2**  Dd and beta 2 microglobulin  [ **B2M** ]  genes, while being ineffective in the cell clone resistant to both types of IFNs.  Ref: 1442172 J Biol Regul Homeost Agents< MedlineTA>J Biol Regul Homeost Agents, 2002
- Mutant mice with a defined genetic defect in the beta 2 microglobulin  [ **B2M** ]  beta 2m or the  **H2**  I A beta chain, which are virtually devoid of functional CD8 or CD4 alpha beta T cells, respectively, were employed for analyzing immune mechanisms involved in acquired resistance against Listeria monocytogenes.  Ref: 7726898 J Immunol, 1994
- The  **H2**  complex, a transmembrane protein, consists of heavy and light chains, the latter is named beta 2 microglobulin  [ **B2M** ] .  Ref: 3515467 Ren Physiol, 1986
- Treatment with IFN gamma and TNF alpha caused 2 to 3 fold enhancement of H 2K and beta 2 microglobulin  [ **B2M** ]  promoter activity, as well as a normalization TNF alpha treatment or enhancement IFN gamma treatment of  **H2**  membrane expression.  Ref: 2517949 J Immunogenet< MedlineTA>J Immunogenet, 1986
- The effect of interferons IFNs type I alpha beta and type II gamma on the stimulation of  **H2**  Dd class I and beta 2 microglobulin  [ **B2M** ]  genes transcription was analysed in IFN sensitive w.t. and IFN resistant Friend erythroleukemia cells FLC.  Ref: 1442172 J Biol Regul Homeost Agents< MedlineTA>J Biol Regul Homeost Agents, 1986
- Association of the mouse major histocompatibility complex MHC class I heavy chain  **H2**  Kb with mouse beta 2 microglobulin  [ **B2M** ]  beta 2m was studied in an in vitro translation system.  Ref: 8500526 Eur J Immunol, 1993
- Genetically engineered mice with a targeted disruption in the beta 2 microglobulin  [ **B2M** ]  beta 2 m gene or the  **H2**  I A beta chain A beta which lack functional CD8 or CD4 T cells, respectively, were used to assess the role of T cell subsets in Brucella abortus infection.  Ref: 7589125 Eur J Immunol, 1995
- Transcriptional induction of  **H2**  class I antigens and beta 2 microglobulin  [ **B2M** ]  by interferon gamma in interferon sensitive and interferon resistant Friend leukemia cells.  Ref: 1442172 J Biol Regul Homeost Agents< MedlineTA>J Biol Regul Homeost Agents, 1995
- The results reported in the present paper suggest that the IFN gamma is able per se to increase the expression of  **H2**  Dd and beta 2 microglobulin  [ **B2M** ]  genes.  Ref: 1442172 J Biol Regul Homeost Agents< MedlineTA>J Biol Regul Homeost Agents, 1995
- Type I IFN enhances the expression of  **H2**  Dd and beta 2 microglobulin  [ **B2M** ]  genes in w.t.  Ref: 1442172 J Biol Regul Homeost Agents< MedlineTA>J Biol Regul Homeost Agents, 1995
